# Influenza-Associated Urticaria Multiforme Mimicking Serum Sickness in a Toddler: A Diagnostic Challenge

**DOI:** 10.7759/cureus.109557

**Published:** 2026-05-24

**Authors:** Iffa Ahmad, Adina Ahmed, Dylan Grau, Judith Cornely

**Affiliations:** 1 Osteopathic Medicine, Nova Southeastern University Dr. Kiran C. Patel College of Osteopathic Medicine, Fort Lauderdale, USA; 2 Pediatrics, Broward Health Medical Center, Fort Lauderdale, USA

**Keywords:** acute annular urticaria, diagnostic challenge, febrile rash, influenza a, pediatric dermatology, serum sickness–like reaction, urticaria multiforme

## Abstract

Urticaria multiforme (UM) is a benign hypersensitivity reaction of early childhood characterized by transient annular urticarial plaques with dusky centers and acral edema. Although self-limited, UM is frequently misdiagnosed as serum sickness-like reaction (SSLR), erythema multiforme (EM), or urticarial vasculitis due to overlapping features such as fever, edema, and inflammatory laboratory abnormalities. Diagnostic uncertainty may therefore lead to extensive investigations, hospitalization, and unnecessary systemic corticosteroid therapy.

A three-year-old male with a history of atopic dermatitis presented with decreased oral intake, lethargy, fever, and intermittent erythematous wheals associated with swelling of the feet and extremities. Initial evaluation revealed influenza A (H3) infection, thrombocytosis, and mildly elevated C-reactive protein. Extensive diagnostic evaluation, including multiple imaging studies and laboratory investigations, was largely unremarkable except for preserved complement levels. Due to concern for a serum sickness-like reaction, systemic corticosteroids were initiated. The dermatology consultation subsequently favored urticaria multiforme based on the transient nature of lesions lasting less than 24 hours, absence of mucosal involvement or palpable purpura, preserved complement levels, and progressive clinical improvement. The patient was managed with antihistamines and supportive care, with complete resolution of fever, edema, and urticarial lesions prior to discharge after a hospital course of six days.

This case highlights influenza-associated urticaria multiforme presenting with fever and mild inflammatory marker elevation, closely mimicking immune complex-mediated disease. Recognition of lesion duration, morphology, and complement preservation is essential for accurate diagnosis and to prevent unnecessary immunosuppressive therapy.

## Introduction

Urticaria multiforme (UM), also referred to as acute annular urticaria of childhood, is a benign hypersensitivity reaction that predominantly affects infants and young children, most commonly between four months and four years of age. First described as a distinct pediatric dermatologic entity by Shah KN and colleagues in 2007, UM represents a morphologic subtype of acute urticaria rather than a separate disease process [1]. Clinically, it is characterized by large annular or polycyclic, blanchable wheals with dusky or ecchymotic appearing centers accompanied by acral edema involving the hands, feet, and occasionally the face [1,2]. The eruption often appears dramatic and may raise concern for more serious inflammatory disorders such as erythema multiforme, serum sickness-like reaction, urticarial vasculitis, and Kawasaki disease; however, individual lesions are transient and typically resolve within 24 hours without residual pigmentation, purpura, or scarring, one of the most important distinguishing features of histamine-mediated urticaria [1,3].

The pathophysiology of UM is thought to involve a type I hypersensitivity reaction driven by mast cell degranulation and histamine release [3,4]. Viral infections are among the most frequently identified triggers, with respiratory viruses playing a prominent role; influenza in particular has been implicated as a potential precipitating factor through immune activation and cytokine-mediated mast cell degranulation [1,5]. Other reported infectious triggers include adenovirus, Epstein-Barr virus, Mycoplasma pneumoniae, streptococcal infections, and upper respiratory viral illnesses. Non-viral triggers include medications such as amoxicillin, cephalosporin, and macrolide antibiotics, as well as immunizations [1,5]. Because viral illnesses are common in early childhood, UM is often observed in otherwise healthy toddlers presenting with acute febrile illness. Systemic findings are typically mild and may include fever, transient acral edema, and pruritus, while laboratory abnormalities, when present, are generally nonspecific and reflect an underlying inflammatory response rather than primary immune complex disease [1,6].

Despite its benign course, urticaria multiforme frequently presents a diagnostic challenge because its annular morphology, acral edema, and associated fever may mimic more serious inflammatory disorders, including erythema multiforme (EM), serum sickness-like reaction (SSLR), urticarial vasculitis, and Kawasaki disease [2,7]. Distinguishing features include the transient nature of UM lesions, which resolve within 24 hours without residual pigmentation or purpura; preserved complement levels; and absence of significant mucosal involvement [1,3]. In contrast, EM typically presents with fixed target lesions and mucosal involvement, SSLR is commonly associated with medication exposure and systemic symptoms such as arthralgias, and urticarial vasculitis presents with painful lesions persisting longer than 24 hours with possible complement consumption [5,7-10].

Diagnostic confusion may lead to extensive laboratory testing, multiple imaging studies, subspecialty consultations, and exposure to systemic corticosteroids or other immunosuppressive therapies. Careful attention to lesion morphology, duration, and associated systemic findings is therefore critical for distinguishing UM from immune complex-mediated disease and preventing unnecessary interventions. Lesion resolution within 24 hours, absence of mucosal involvement or palpable purpura, preserved complement levels, and a benign clinical course remain key diagnostic features [1,2].

We present a case of influenza-associated urticaria multiforme in a febrile three-year-old male with decreased oral intake, intermittent urticarial plaques, acral edema, and mild inflammatory laboratory abnormalities. The patient underwent extensive diagnostic evaluation due to concern for systemic inflammatory disease and was initially treated for a possible serum sickness-like reaction. Dermatologic evaluation ultimately confirmed urticaria multiforme based on the transient nature of the lesions and preserved complement levels. This case highlights how influenza-associated UM with mild inflammatory marker elevation can obscure diagnostic clarity and emphasizes the importance of recognizing its characteristic clinical features to avoid unnecessary investigations and immunosuppressive therapy.

## Case presentation

A three-year-old male presented with acute decreased oral intake, increased somnolence, and reduced activity from baseline. He had a history of atopic dermatitis treated intermittently with topical corticosteroids and calcineurin inhibitors, without an active eczema flare at presentation. The evening prior, he was playful and eating normally. On the day of presentation, he developed poor oral intake and progressive fatigue and was noted to have a moderate-grade fever at his pediatrician’s office, prompting referral to the emergency department (ED). Caregiver history was notable for bilateral lower extremity pain and right foot swelling starting the day prior, as well as intermittent, waxing, and waning erythematous, raised wheals one week ago. There was no history of prior trauma, as ruled out by ED in the context of right-hand and foot swelling. There was no family history of atopy or exposure to allergens or medications. A prior ED visit documented right-hand and right-foot swelling of unclear etiology following a possible fall.

In the ED, chest radiograph, abdominal and pelvic computed tomography (CT) with contrast, and ultrasounds of the appendix, intussusception, and testicles were largely unremarkable, aside from moderate stool burden and mild right lower quadrant adenitis on a kidney, ureter, and bladder (KUB) scan. On physical exam, he had mild periorbital puffiness with faint conjunctival erythema, cracked lips, dry tongue with white coating, and tense edema of the right foot and hand. He held his lower extremities in slight flexion and was tender to palpation below the knees. He developed annular, polycyclic urticarial plaques involving the trunk and extremities. Individual lesions resolved within 24 hours and migrated to new locations with periods of complete lesion resolution between episodes. There was no mucosal ulceration, blistering, necrosis, or palpable purpura noted at any point. No cervical or generalized lymphadenopathy was observed.

Laboratory studies were notable for influenza A (H3) positivity, sodium 134 mmol/L, bicarbonate 17 mmol/L, AST 56 U/L, relative neutrophilia (76.8%), and normal total leukocyte count without eosinophilia (Table 1). Platelets were elevated at 440 ×10³/µL and C-reactive protein (CRP) was elevated at 1.38 mg/dL (Table 2). Urinalysis showed mild proteinuria and ketonuria (Table 3). Lipase and creatine kinase were normal. Blood and urine cultures were obtained. He developed transient facial swelling following CT imaging, which improved with diphenhydramine. Supportive care with intravenous (IV) fluids, oseltamivir, ibuprofen, and diphenhydramine as needed (PRN) was initiated.

**Table 1:** Comprehensive Metabolic Panel on Admission

Comprehensive Metabolic Panel (CMP)	Result	Reference Value
Sodium	134 mEq/L	135–145 mEq/L
Bicarbonate	17 mmol/L	23 - 29 mmol/L
Creatinine	0.6 mg/dL	0.8-1.2 mg/dL
AST	56 U/L	12-37 U/L

**Table 2:** C-Reactive Protein (CRP) and Platelets on Admission

Test	Admission	Reference Value
C-reactive protein	1.38 mg/dL	≤ 0.80 mg/dL
Platelets	440 x 10³/µL	140-400 x 10³/µL

**Table 3:** Urinalysis on Admission and Recheck

Urinalysis	(Admission)	Day 3 (Recheck)	Reference Value
Proteins	30.0 mg/dL	0 mg/dL	0 mg/dL
Ketones	40.0 mg/dL	0 mg/dL	0 mg/dL

On day two of admission, the patient continued to have intermittent fevers with a spike of 39.3°C. Physical examination revealed transient erythema of the cheeks and arms, swelling of the right elbow and left foot, and dry lips without mucosal erythema. No desquamation, cervical lymphadenopathy, or persistent conjunctival injection was observed. Given concern for inflammatory etiologies, pediatric rheumatology and dermatology were consulted. Antinuclear antibody (ANA), anti-double-stranded DNA (anti-dsDNA), complement component 3 (C3), complement component 4 (C4), antistreptolysin (ASO) titers, and mycoplasma antibodies were ordered. An electrocardiogram (ECG) and an echocardiogram were also ordered.

On day three of admission, clinical status improved overall, though intermittent urticarial eruptions persisted. Rheumatology recommended increasing IV methylprednisolone to 10 mg/kg/day and additional laboratory evaluation, including total hemolytic complement (CH50), antineutrophil cytoplasmic antibody (ANCA), Epstein-Barr virus (EBV) serologies, and immunoglobulin levels. C3, C4, and ASO titers were unremarkable (Table 4). Repeat urinalysis was negative for protein and ketones. Dermatology favored a diagnosis of urticaria multiforme versus a serum sickness-like reaction and did not recommend a biopsy. Caregivers were counseled regarding the typically self-limited nature of the condition. Topical hydrocortisone 2.5% was recommended for recurrent lesions, along with routine emollient therapy for underlying atopy.

**Table 4:** Important Values Distinguishing Urticaria Multiforme and Serum Sickness-Like Reaction (SSLR)

Test	Admission	Day 2	Day 3	Day 4	Reference Value
C3 complement		119 mg/dL			80-170 mg/dL
C4 complement		34.50 mg/dL			14-44 mg/dL
EBV VCA IgG				641 U/mL	<18 U/mL
EBV EBNA Ab				456 U/mL	<18 U/mL

On day four of admission, the caretaker reported overnight facial and scalp pruritus that resolved by morning. Extremity swelling and rash had significantly improved. High-dose methylprednisolone was continued for a planned 2-3 day course. Immunoglobulin levels (IgA, IgG, IgM) were normal. Gastrointestinal pathogen polymerase chain reaction (GI PCR) testing and echocardiogram were unremarkable. The patient experienced brief recurrent hives overnight. Rheumatology recommended completion of a third day of high-dose steroids followed by an outpatient prednisone taper with a shift to fexofenadine 30 mg/5 mL suspension as needed. The ECG demonstrated ST elevation interpreted as likely early repolarization. Additional laboratory evaluations, including CH50, ANCA, and Immunoglobulin E (IgE), were unremarkable. EBV serologies were consistent with prior infection.

On day five of admission, the patient remained afebrile with resolution of extremity swelling and minimal urticarial activity in the preceding 24 hours. He was cleared by rheumatology and discharged on a prednisone taper (5 mL twice daily for seven days, then 5 mL daily for seven days), fexofenadine as needed, famotidine for 10 days, and completion of oseltamivir for one day. Outpatient follow-up was arranged with pediatrics, pediatric dermatology, and rheumatology.

## Discussion

Urticaria multiforme (UM) is frequently misinterpreted as an immune complex-mediated disorder because of its annular morphology, acral edema, and associated fever [1,2]. These features may resemble erythema multiforme (EM), serum sickness-like reaction (SSLR), urticarial vasculitis, Kawasaki disease, or viral exanthem, often prompting extensive diagnostic evaluation. In this case, fever, extremity swelling, abdominal complaints, and inflammatory laboratory abnormalities initially raised concern for systemic inflammatory disease. However, the transient nature of the lesions (<24 hours), absence of mucosal involvement or palpable purpura, preserved complement levels, and rapid clinical improvement favored a diagnosis of urticaria multiforme. Key distinguishing clinical features between UM and its common mimickers are summarized in Table 5.

**Table 5:** Clinical Features Distinguishing Urticaria Multiforme From Similar Pediatric Dermatologic Conditions

Feature	Urticaria Multiforme	SSLR	EM	Urticarial Vasculitis
Lesion duration	<24 hours	Days	Fixed	>24 hours
Mucosal involvement	Rare	Rare	Common	Rare
Arthralgia	Rare	Common	Rare	Possible
Complement levels	Normal	May decrease	Normal	Often decreased
Residual pigmentation	No	No	Possible	Common
Trigger	Viral infections	Medications	HSV/infections	Autoimmune disease

The transient duration of individual lesions remains one of the most reliable distinguishing features of UM. Urticarial wheals typically resolve within 24 hours without residual dyspigmentation or scarring, reflecting a histamine-mediated process limited to the superficial dermis [1,3]. In the present case, the child’s eruption consisted of migratory, blanchable plaques that appeared and resolved within hours without residual skin changes, supporting a histamine-mediated urticarial process rather than vasculitis or epidermal injury.

Serum sickness-like reaction (SSLR) remained an important diagnostic consideration because the patient presented with fever, edema, and annular urticarial plaques. However, there was no recent medication exposure, arthralgias, lymphadenopathy, or hypocomplementemia, making immune complex-mediated disease less likely [5,8,11]. Similarly, Kawasaki disease was considered because of fever and mucocutaneous findings, though the patient did not meet diagnostic criteria due to limited fever duration and absence of persistent conjunctivitis, lymphadenopathy, desquamation, or significant mucosal involvement.

The presence of fever and mild inflammatory laboratory abnormalities initially complicated the diagnostic evaluation. The patient demonstrated mild thrombocytosis, relative neutrophilia, and a modestly elevated C-reactive protein (CRP) level during hospitalization. However, these findings are likely attributable to the concurrent viral infection rather than a primary systemic inflammatory disorder. Viral infections are implicated in approximately 60-80% of acute urticaria cases in children, with respiratory viruses, such as influenza, representing the most common infectious triggers [1,6,10]. In this case, the patient tested positive for influenza A (H3) on the respiratory viral panel, providing a plausible antigenic stimulus for mast cell activation and histamine release, supporting influenza as the likely precipitating trigger. Respiratory viral infections have been shown to trigger urticarial eruptions through immune activation and cytokine-mediated mast cell degranulation [10]. Importantly, mild elevations in inflammatory markers should be interpreted cautiously, as they may reflect viral immune activation rather than immune complex-mediated pathology. Table 6 shows the reportable triggers of urticaria multiforme.

**Table 6:** Reportable Triggers of Urticaria Multiforme

Reportable Triggers	Examples
Viral	Influenza, adenovirus, EBV
Bacterial	Streptococcus, Mycoplasma
Medications	Amoxicillin, Cephalosporin
Vaccinations	Measles, mumps, rubella (MMR), Diphtheria, tetanus, and acellular pertussis (DTaP), influenza vaccine

The extensive diagnostic workup performed in this case further illustrates how the dramatic presentation of UM can lead to evaluation for multiple systemic conditions. The patient underwent broad imaging, including chest radiography, abdominal and pelvic computed tomography (CT) imaging, and multiple abdominal ultrasounds, largely to evaluate abdominal pain and exclude surgical pathology such as intussusception or appendicitis. Laboratory testing included autoimmune and rheumatologic markers, complement levels, viral studies, and immunoglobulin panels. These investigations ultimately remained unremarkable, aside from the positive influenza test and nonspecific inflammatory markers. The absence of hypocomplementemia, negative autoimmune serologies, and benign imaging results further supported a non-immune complex-mediated process.

Pathophysiologically, UM represents a type I hypersensitivity reaction involving mast cell activation and histamine release within the superficial dermis [3,4]. This process may occur through IgE-dependent or non-IgE-dependent pathways and results in localized dermal edema and vasodilation without vascular injury or immune complex deposition [3,4]. In contrast, SSLR and urticarial vasculitis involve immune complex formation and complement activation, leading to endothelial damage and a more prolonged inflammatory response [4,9]. The preserved complement levels observed in this patient are therefore consistent with the histamine-mediated mechanism underlying UM and help distinguish it from immune complex-mediated disease.

Management of UM is primarily supportive. Antihistamines, specifically Fexofenadine 30 mg/5 mL every 12 hours as needed, remain the cornerstone of therapy, as they directly counteract histamine-mediated vascular permeability and pruritus [3]. In most cases, symptoms resolve within several days, without the need for systemic immunosuppression. Although clinical improvement occurred following corticosteroid administration, the self-limited course and characteristic lesion morphology favored urticaria multiforme, making it difficult to determine whether corticosteroids significantly altered disease progression. Additionally, interpretation of immunologic laboratory studies, particularly immunoglobulin E (IgE) levels, should be approached cautiously because some samples were obtained after initiation of systemic corticosteroid therapy, which may influence inflammatory and immunologic markers. The patient ultimately improved rapidly with supportive care, antihistamines, and a tapered prednisone regimen. This highlights the importance of early dermatologic consultation when atypical urticarial eruptions occur in pediatric patients.

This case also highlights several clinically relevant diagnostic considerations. Fever should not exclude urticaria multiforme, particularly in the setting of concurrent viral infection such as influenza [1]. Similarly, mild elevations in inflammatory markers may reflect viral immune activation rather than primary systemic inflammatory disease. Complement testing may provide an additional diagnostic adjunct when distinguishing histamine-mediated urticaria from immune complex-mediated disorders [3,9]. However, the most reliable distinguishing feature remains the transient duration of individual lesions, which typically resolve within 24 hours without residual pigmentation or purpura [1,2]. Recognition of these characteristic findings may help reduce unnecessary diagnostic evaluation and treatment escalation. 

Systemic corticosteroids are generally not required in the management of urticaria multiforme and may expose pediatric patients to avoidable adverse effects, including behavioral changes, hyperglycemia, hypertension, and immunosuppression [3,12]. Increased awareness of UM among pediatricians, emergency physicians, and hospitalists may therefore improve diagnostic accuracy and reduce unnecessary treatment escalation. Given the high frequency of viral illnesses in early childhood, clinicians should maintain a high index of suspicion for urticaria multiforme when evaluating febrile toddlers presenting with transient annular eruptions and acral edema.

## Conclusions

Influenza-associated urticaria multiforme can closely resemble serum sickness-like reactions and other immune complex-mediated conditions in febrile toddlers, particularly when accompanied by mild inflammatory marker elevation. However, urticaria multiforme (UM) remains a benign, histamine-mediated hypersensitivity reaction characterized by transient lesions resolving within 24 hours, absence of mucosal involvement, and preserved complement levels. Recognition of these distinguishing clinical features is critical to avoid unnecessary hospitalization and extensive diagnostic evaluation, including autoimmune and rheumatologic panels, complement levels, viral studies, immunoglobulin panels, multiple imaging modalities, and systemic corticosteroid exposure. This case reinforces that fever and mild laboratory abnormalities do not exclude urticaria multiforme, especially in the setting of concurrent viral infection. Careful attention to lesion duration and morphology remains the most reliable diagnostic approach. Increased clinician awareness of this entity can improve diagnostic accuracy, promote judicious use of immunosuppressive therapy, and enhance patient safety in pediatric populations.
